# 4291 Quantifying the art of surgical decision-making in total knee arthroplasty

**DOI:** 10.1017/cts.2020.312

**Published:** 2020-07-29

**Authors:** Shady Elmasry, Carl Imhauser, Timothy Wright, Peter Sculco, Cynthia Kahlenberg, Geoffrey Westrich, Michael Cross, David Mayman, Andrew Pearle

**Affiliations:** 1 Hospital for Special Surgery

## Abstract

OBJECTIVES/GOALS: To quantify clinical exam in total knee arthroplasty by answering the following questions: (1) What are the magnitudes of forces applied by surgeons during the varus-valgus exam? (2) Is the choice of tibial insert thickness related to the magnitude of the applied forces? (3) How accurately does a surgeon estimate the gaps in the varus-valgus exam? METHODS/STUDY POPULATION: Three cadaveric knees were implanted with standard TKA trial implants. Four pliable force sensors were wrapped around the foot and ankle of each cadaver to measure the push-pull forces applied during the varus-valgus exam. Six surgeons with varying experience independently conducted a varus-valgus exam in extension and flexion and reported the gaps that they observed. Motion capture was used to measure the gaps between femur and tibia by placing cluster of reflective markers on femur and tibia. Subsequently, each surgeon chose the tibial insert that they thought best fit each knee. The measured peak applied forces were related to the insert thickness and the measured gaps were compared to the observed gaps by surgeons. Since insert thickness was in 1 mm increments, 1 mm gap error was considered a meaningful difference. RESULTS/ANTICIPATED RESULTS: The peak forces varied among surgeons for each cadaver. In cadaver one, the peak forces in varus and valgus in extension were 48±20 and 20±12 N, and in flexion were 27±14 and 8±11 N. Peak forces in cadavers two and three were similar; in varus and valgus in extension, 24±14 and 35±10 N, and in flexion, 23±12 and 20±10 N, respectively. It was observed that the larger the valgus force in extension, the thinner was the inserts choice (β = −0.08 mm/N, p = 0.012). In extension, the difference between estimated gaps and measured gaps was > 1 mm for 36% of all assessments and 91% of gaps were underestimated. Only one measure, however, was underestimated by > 2 mm. In flexion, gap estimates were > 1 mm for 35% of all measurements and 59% of all measurements were overestimated. Four measures were overestimated, and one was underestimated by > 2 mm. DISCUSSION/SIGNIFICANCE OF IMPACT: We found that the applied forces varied among surgeons and a negative association between insert thickness and forces in extension valgus exam. We also found that error in gap estimates among surgeons was > 1 mm a third of the time and that underestimation is more common in full extension, which may lead to using smaller inserts that affect knee stability. CONFLICT OF INTEREST DESCRIPTION: The corresponding author has no COI but my coauthors had the following COI:

1. Royalties from a company or supplier: *Zimmer; Stryker; Exactech, Inc; Lima; Mathys Ltd.*
2. Speakers bureau/paid presentations for a company or supplier *Acelity; Flexion Therapeutics; Smith & Nephew; Exactech, Inc; Mallinckrodt Pharmaceuticals; Stryker.*
3. Paid consultant for a company or supplier *Acelity; DePuy Synthes; Exactech, Inc; Flexion Therapeutics; Intellijoint; Smith & Nephew; Zimmer; Stryker*
4. Stock or stock options in a company or supplier *Imagen; Insight Medical; Intellijoint; Parvizi Surgical Innovation; OrthAlign; Orthobond.*
5. Research support from a company or supplier as a Principal Investigator *Acelity; Exactech, Inc; Intellijoint; Smith & Nephew; Mallinckrodt Pharmaceuticals; Stryker; Lima.*
6. Royalties, financial or material support from publishers (The following conflicts were disclosed) *Exactech, Inc.*
7. Medical/Orthopaedic publications editorial/governing board *Bone and Joint Journal 360; Journal of Orthopaedics and Traumatology; Techniques in Orthopaedics.*
8. Board member/committee appointments for a society Knee Society; *Eastern Orthopedic Association.*

